# Supplementary material for: Learning curve and surgical outcome of robotic assisted colorectal surgery with ERAS program
Source: Sci Rep. 2022 Nov 29;12:20566. doi: 10.1038/s41598-022-24665-w (PMC9709162; doi:10.1038/s41598-022-24665-w)
Supplement: Supplementary file 1 — Supplementary Information 1. [file 41598_2022_24665_MOESM1_ESM.zip › LSC Raw data-Ñ[▒K/135/O_ñw╝╨░O▒Kñσ.pdf]

正式摘要(電子簽章：000000223723717)

W83 / W83

臺 中 榮 民 總 醫 院

Operation Note

[redacted]

- 手術日期：2021/08/27
- 手術醫師：VS 蔣鋒帆，R 陳璿夫，R 洪浚嚴
- Anesthesia：GE
- Position：Lithotomy
- Aseptic agents：Soap

Diagnosis

- Pre-operative diagnosis : Malignant tumor of rectum FOR COMBINE RESECTION
- Post-operative diagnosis : Ditto., S/P laparoscopic LAR with anastomosis+ laparoscopic S5, S6 subsegment hepatectomies

Surgical Indication

- for curative resection

Operation

- 1. Laparoscopic low anterior resection with loop ileostomy
  2. Intraoperative colonoscopy：(-)
  3. High ligation (+)：At IMA root. IMV and left colic artery were also ligated.
  4. EndoGIA (60-4.1；Green) X4 + EEA (29 mm) were used
  5. Proximal cut end: 15 cm, distal cut end: 2 cm
  6. Loop ileostomy was created and fixed over RLQ of abdomen (About 20 cm prior to cecum).
  7. One 19 Fr. hemovac tube was placed from LLQ of abdomen and inserted into the pelvic cavity
  8. The colon was taken down from the splenic flexure site

Finding

- 1. A 3 x 3 cm, ulcerative tumor at lower rectum. (DRE：Finger tip)
  2. No other gross tumor nor polyps was found
  3. There were obvious visible liver metastasis, S5, S6 under laparoscope and intraoperative sonography; no visible peritoneal seeding.
  4. No obvious ascites was noted over the pelvic area.
  5. Bilateral doughnuts：intact；Air leak test: Done and no air leak was found

Procedure

- 1. General endotracheal anesthesia was induced and then the patient was placed in modified Trendelenburg
  lithotomy position. Preoperative antibiotics were given. A Foley catheter and a nasogastric tube were placed. The abdomen was prepared and draped in standard sterile fashion.
  2. A small vertical incision was made right next to the umbilicus and a 12 mm trocar was inserted into the
  peritoneal cavity by open method. Peumoperitoneum was established with CO2 inflation. Laparoscopy was inserted
  and then another 12 mm trocar was inserted over RLQ. Extra three 5 mm trochars were inserted over RUQ, LUQ and
  LLQ of abdomen. The operative findings were mentioned as above.
  3. The small bowel was inspected and retracted to the right by grasper. The sigmoid colon was then held by

- grasper and the peritoneum overlying the mesentery was then scored with electrocautery. The inferior mesentery
  artery was identified, ligated below the left colic artery with endoclips, and transected, so was inferior

  mesentery vein. Dissection was done above the Gerota's fascia and toward the peritoneal attachments.
  4. The arcade vessels to the sigmoid colon were also divided. Distal descending colon and upper rectum were mobilized.
  5. The colon was mobilized from retroperitoneum overlying the sacral promontory and into the pelvis. The incision was extended laterally and medially across the peritoneal reflection.
  6. The mesorectum was then mobilized using sharp dissection over the level of pelvic floor muscle. Then distal descending colon and rectum were mobilized as possible.
  7. DRE was performed at the same time to make sure the desired resection line is adequate.
  8. Totally 4 linear staplers (EndoGIA 60-4.1) were used to divide the lower rectum.
  9. Incision was further extended from the LLQ 5 mm trochar wound. The proximal colon was then taken out for carefully inspection, and to make sure it has an excellent blood supply. The mesenteric fat was gently cleared from the terminal 2-3 mm of the bowel ends.
  10. The field was isolated with moist pad and the bowel opened and sized. It was determined that a 29 mm circular stapler could be accommodated. A pursestring suture of 2-0 prolene was placed, the anvil was inserted, and then the pursestring suture was tied.
  11. The anal canal was irrigated with distil water and then the circular stapler was introduced through the anus and pierced through the mid-portion of the stapler line.
  12. The proximal bowel with anvil in place was engaged with the circular stapler and the stapler was then fired. Two complete donuts were obtained and the distal one was submitted to pathology for margin confirmation.
  13. The anastomosis was tested for leaks with irrigation solution and air introduced through the rectum and was found to be patent and intact.
  14. Laparosopic S5, S6 subsegment hepatectomies was performed by GS Chief Doctors
  15. The possible bleeder was then checked thoroughly.
  16. A small new incision wound was made above the LLQ extended wound for inserting the 19 Fr. hemovacdrainage tube, which was placed into the pelvic.
  17. The ADHESION BARRIER FILM was then placed below the LLQ extended wound.
  18. The desired terminal ileum (20 cm prior to the cecum) was selected. A window was created under the ileum by dividing the mesentery adjacent to the bowel wall with electrical cautery carefully. Then it was looped via Silicone Vessel loops.
  19. The wound over the RLQ trochar site was further extended to serve as the ostomy site.
  20. The terminal ileum was then dragged out from the ostomy site carefully and a plastic bridge (Rod) was placed via the window.
  21. The fascia of midline wound was closed with interrupted sutures via 1-O Vicryl. The LLQ extended wound was further closed with interrupted method via Num. 1 Vicryl.
  22. After irrigation the wound with N/S, the skin was closed with interrupted 3-0 nylon (Local anesthetic agent was injected below the wound site.), and the smaller wound sites were covered with Heraderm. The larger wound, which was closed to the drainage tube site, was covered with gauze instead.
  23. The ileostomy was matured with multiple interrupted mucocutaneous suture via 4-0 Monocryl.
  24. The ileostomy was well formed and viable. An ostomy bag was placed.
  25. The patient tolerated the procedure well and was brought to the POR in stable condition
- Estimated blood loss: minimum
- 器械數量: 正確
- 紗布數量: 正確

Image

- PAINT
  - abdomen

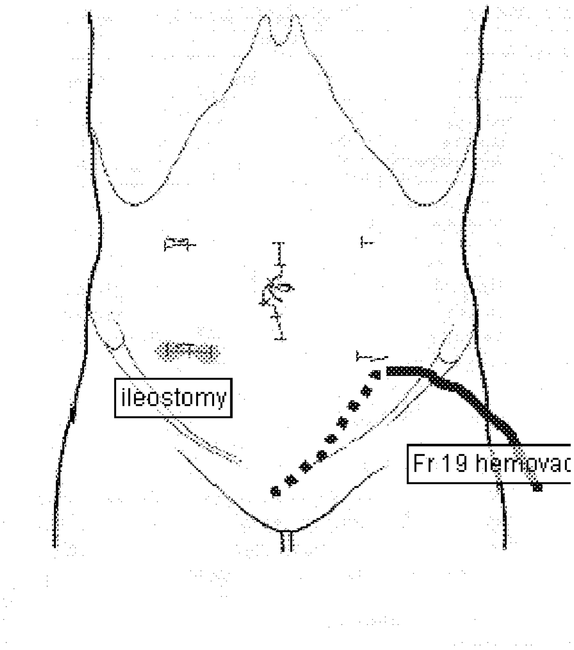

- colon & rectum

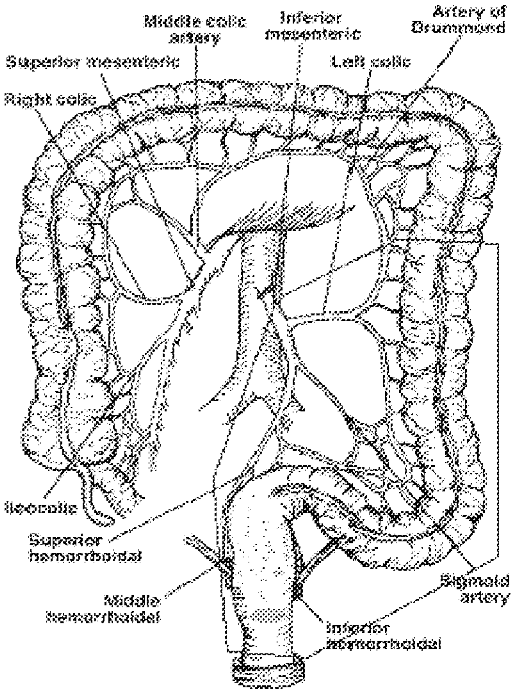

主任 2062F蔣鋒帆 / 醫師 2310K陳璿夫

正式摘要(電子簽章：000000223723717)

W83 / W83

doctorRank:B
